# Differences in the Levels of Physical Activity, Mental Health, and Quality of Life of Elderly Koreans with Activity-Limiting Disabilities

**DOI:** 10.3390/ijerph16152736

**Published:** 2019-07-31

**Authors:** Kyujin Lee, Wi-Young So

**Affiliations:** 1 College of Health and Human Development, California State University, Northridge, CA 91330, USA; 2 College of Humanities and Arts, Korea National University of Transportation, Chungju-si 27469, Korea

**Keywords:** elders with disabilities, mental health, physical activity, quality of life

## Abstract

*Purpose*: This study aimed to analyze the differences in physical activity, mental health, and quality of life between the elderly without activity-limiting disabilities and in elderly with activity-limiting disabilities, stratified by medical category. *Methods*: A total of 1504 elders aged 65–80 who responded to the 2016 Korea National Health and Nutrition Examination Survey were the participants in this study. They were divided into two groups, 303 elders with disabilities who responded to the survey saying they have active limiting disabilities, and 1201 elders without disabilities. The questionnaire reflected the criteria of a survey developed by the American College of Sports Medicine (ACSM) to measure the physical activity of elders. Patient Health Questionnaire-9 (PHQ-9) and EuroQol-5 Dimension (EQ-5D) were used to measure participants’ mental health and quality of life, respectively. Analysis of covariance (ANCOVA) adjusted for age, sex, marital status, notional basic livelihood, recipient status, and personal income was conducted for data analysis after the normal distribution was confirmed. *Results*: Significant differences between elders with disabilities and those without disabilities were seen in terms of physical activity levels (*p* < 0.01), stress, and depression (*p* < 0.001), and in all five domains measuring the quality of life (*p* < 0.05). The ANCOVA revealed significant differences in motor ability (*p* < 0.01) and pain/discomfort (*p* < 0.05) relating to the quality of life among the elders with activity-limiting disability depending on the medical category. *Conclusions*: The results could provide basic data regarding the degree of physical activity, mental health, and quality of life according to activity-limitation disability status and medical category. From the findings, elders with disabilities are clearly more exposed to risk in the areas of physical activity, mental health, and quality of life. Differences in quality of life among elderly with disabilities were also seen based on medical category. Our findings suggest that research into elders with disabilities should consider the implications of these results.

## 1. Introduction

The 2015 Korean National Statistics Office Survey found that 14.5% of the Korean population was over 65 years. It has been predicted that post-2025, more than 20% of the elderly will come under the category of the super-aged [1]. With the rapid aging of society comes a parallel increase in the number of elders with disabilities. Although the number of Koreans under 50 years old with disabilities is decreasing, the trend shows a continuous increase in those over 65 years, from 38.8% in 2011 to 43.3% in 2014, and 46.6% in 2017 [2]. Since there are already Korean elders with disabilities in the super-aged category, finding resources to help them with their physical disabilities and improve their overall quality of life have become important issues [3].

Elderly with disabilities can be divided into two groups: aging with disability and disability with aging. These two groups are commonly called “elders with disabilities.” They experience limitations in their daily lives regardless of the reason for or type of disability [4,5]. Elders with disabilities are a vulnerable class; besides the disadvantage of aging, they also have to cope with disability [4,6]. In addition, they may not be able to receive proper treatment or intervention for any chronic diseases or for their specific disability, which may result in further health problems [7,8]. Elders with activity-limiting disabilities may have a higher risk of chronic disease and comorbidities along with disease-related symptoms such as high blood pressure, low strength, and a poor mental status and quality of life [3]. There is a higher possibility of elders with disabilities being exposed to health-risk factors as well as a lesser ability to recover; thus, it is important that they receive active interventions to improve their quality of life [9].

Physical activity behavior is related to risk reduction of chronic disease conditions, functional limitations of the elderly, and quality of life [10,11]. Disabled elderly who experience activity restriction find it hard to enjoy the positive effects of physical activity with regard to quality of life as participation in physical activity is difficult for them [12,13]. The possibility of differences in quality of life, physical activity, and mental health according to the medical category that induces disability has been suggested [12], but there is as yet no general conclusion regarding the effects of different medical categories of disability on these areas as large-scale studies related to this issue have not yet been conducted.

To help them maintain their physical health behaviors and to prepare a program to enhance the quality of their lives, it is important to consider the physical activity, mental health, and quality of life of elders with disabilities [14,15,16]. Previous studies have focused on evaluating physical activity [17], mental health [18], and quality of life [19] among elders to recommend the amount of physical activity required of them. The three areas have also been examined among elders as a dependent factor [17,18,19] to check if they are correlated to each other [20,21,22]. Additionally, some researchers [20,21,22] have investigated methods to improve sedentary behavior among elderly with specific disabilities (e.g., intellectual disabilities or disabilities associated with diabetes mellitus). However, studies are lacking regarding the differences in physical activity, mental health, and quality of life among the disabled elderly stratified by medical category, and only limited information exists to evaluate elders with activity-limiting disabilities in terms of these aspects of their lives.

This study provides the basic data needed for promoting the health of elders with disabilities by understanding their degrees of physical activity, mental health, and quality-of-life status, and comparing and analyzing the differences between the different disabilities according to the medical category to which they belong. The aim of the study is, therefore, to compare and analyze these differences based on activity-limiting disability and medical category. We hypothesized that the physical activity, mental health, and quality-of-life status of elders with activity-limiting disabilities would be different from that of elders without disabilities. Furthermore, we hypothesized that for elders with disabilities, these areas would vary depending on disability status and medical category.

## 2. Methods

### 2.1. Research Design

This is a descriptive survey study based on the 2016 Korea National Health and Nutrition Examination Survey (KNHANES) aimed at analyzing the differences in the physical activity, mental health, and quality of life of the elderly based on the activity limitation of the disability (meaning a difficulty encountered by an individual in executing a task or action) [23], the disability status, and the medical category [24]. The Korea Centers for Disease Control and Prevention have been conducting the KNHANES cross-sectional nationwide surveys since 1988, using a stratified multistage cluster-sampling design to derive a nationally representative sample. The questionnaire has been verified for reliability and validity [24]. This study was conducted in accordance with the Declaration of Helsinki, and all procedures were approved by the ethics committee of the Korean Centers for Disease Control (Institutional Review Board No. 2015-01-02-6C).

### 2.2. Participants

The 2016 KNHANES survey was conducted on 4416 families in 192 survey districts, and 1534 elders aged 65 to 80 (with disabilities = 313, without disabilities = 1221) participated. Activity-limitation disability status was defined as the difficulty encountered by an individual in executing a task or action. Exclusion criteria were: (1) under 65 years old; (2) unclear status regarding the activity-limitation disability; (3) no submission of medical condition; (4) unreliable results from the survey. Elderly with cognitive deficits such as dementia or mental retardation were not excluded. Questionnaires were to be completed with help from family members if participants had difficulty filling out the survey. Of the total 1534 participants, 30 (1.96%; with disabilities = 10; without disabilities = 20) were excluded because they had not completed some or any sections of the questionnaire. Thus, data from 1504 elders aged 65 to 80 years (with disabilities = 303, without disabilities = 1201) were included for the analysis. The KNHANES team also evaluates the health level and health behavior of Koreans every three years. The general characteristics of participants such as age, gender, marital status, marital condition, national basic livelihood recipient status, and personal income were derived from this survey (see Table 1).

### 2.3. Data Collection

The data for this study were drawn from the 2016 KNHANES survey. We evaluated three domains: physical activity, mental health, and quality of life. To evaluate physical activity, participants completed the survey reflecting the ACSM-recommended physical activity criteria. Aerobic physical activity was evaluated by whether or not they participated in such activity for more than 150 min per week, and also by the number of days they walked for at least 10 min per day. Strength exercise was defined as physical activity that forces muscles to work by using resistance, like a dumbbell or one’s own body weight. Strength exercise was evaluated by days participating in strength exercises per week. To evaluate mental health, participants assessed their stress and depression levels using Patient Health Questionnaire-9 (PHQ-9) [25]. Quality of life was measured using the EuroQol-5 Dimension (EQ-5D) comprising questions involving their present status in five domains: motor ability, self-management, daily life, pain/discomfort, and anxiety/depression [26]. The possible answers were “no problem” (level 1), “a moderate problem” (level 2), and “a severe problem” (level 3). In the 2016 KNHANES, activity-limiting disabilities were divided into 24 medical categories (fracture/articulation injury, other injuries, arthritis/rheumatism, heart disease, breathing problems/lung disease/asthma, stroke, diabetes, hypertension, back/neck problems, cancer, tooth and dental disease, vision problems, hearing problems, dementia, depression/anxiety/emotional problems, mental retardation, obesity, old age (people over 65 years no particular reason), renal failure, dizziness, gastrointestinal disturbance, knee/leg pain (except for arthritis), headaches, and other).

### 2.4. Statistical Analysis

Means and standard deviations were calculated for all data and were analyzed using SPSS 21.0 (IBM Corp., Armonk, NY, USA). Frequency analysis was conducted to assess the participants’ characteristics. After normal distribution was confirmed, analysis of covariance (ANCOVA) was conducted to control several confounders (age, sex, marital status, notional basic livelihood, recipient status, and personal income) to examine the differences in physical activity, mental health, and quality of life based on activity limitations corresponding to the disability status of the elders. After a homogeneity of variance test was completed to evaluate normal distribution, ANCOVA with a post-hoc test (Bonferroni) was conducted to control several confounders (age, sex, marital status, notional basic livelihood, recipient status, and personal income) to analyze the differences in physical activity, mental health, and quality of life. The case of an elder with mental retardation (*n* = 1) had to be eliminated because it is impossible to run a Bonferroni test if a group has only one case. Statistical significance was set at *p* < 0.05 for all analyses.

## 3. Results

### 3.1. Differences in Physical Activity, Mental Health, and Quality of Life Among Elders with Activity-Limiting Disabilities and Those without a Disability

Among the 1504 elders (with disabilities = 303, without disabilities = 1201) who completed the physical activity survey, 73 elders with disabilities (24.09%), and 432 elders without disabilities (35.97%) participated in aerobic physical activity (over 150 min a week). In the section on strength exercises per week, 31 elders with disabilities (10.23%) and 215 elders without disabilities (17.90%) reported participating in resistance exercises more than once a week. For the number of days they walked per week (more than 10 min per day), elders with disabilities reported walking on 2.66 ± 1.78 days, and elders without disabilities walked on 3.51 ± 1.82 days. There were significant differences between the two groups of elders in terms of their participation in all three physical activities (*p* < 0.01). In the mental health domain, the sum of the responses from the survey represents increased severity of stress as the number gets closer to 1. Elders with disabilities showed a significant level of stress (2.79 ± 0.94) compared to elders without disabilities (3.20 ± 0.86) (*p* < 0.001). Depression levels based on the PHQ-9 test also showed that elders with disabilities showed a significant difference (two times higher; 6.12 ± 5.81 vs. 2.36 ± 3.64) compared to elders without disabilities (*p* < 0.001). Finally, among the quality-of-life variables, significant differences were seen between the two groups in terms of motor ability (*p* = 0.007), self-management (*p* = 0.013), daily life (*p* = 0.018), pain/discomfort (*p* < 0.001), and anxiety/depression (*p* = 0.021; see Table 2).

### 3.2. Differences in Physical Activity, Mental Health, and Quality of Life Based on Medical Category among Elders with Disabilities

The percentage of activity limitation for each disability type is shown in Table 3. The total number recorded across all medical categories was greater than the total number of elders with disabilities because every participant indicated they had multiple disabilities. The differences among the elders with disabilities in physical activity, mental health, and quality of life based on medical category are shown in Table 4. ANCOVA revealed no significant differences in the physical activity and mental health domains. However, motor ability (F = 2.065, *p* = 0.004) and pain/discomfort (F = 1.809, *p* = 0.021) showed significant differences in the quality-of-life domain. Nevertheless, a post-hoc test (Bonferroni) revealed that the difference in the quality-of-life domain was not statistically significant among medical categories.

## 4. Discussion

The present study aimed to analyze physical activity, mental health, and quality of life among elderly Koreans with and without activity-limiting disabilities, including analysis of those with disabilities by medical category. Data on these areas were obtained from 1504 elders aged 65 to 80 who participated in the 2016 KNHANES.

A decline in physical activity, strength, and ambulatory ability among elders is a general characteristic of frailty. Physical activity participation and fitness maintenance are important factors for the prevention and management of frailty [27,28]. Elders with disabilities find it difficult to engage in physical activity because they face various obstacles. They struggle to maintain an independent lifestyle because there is a greater risk of falling [29]. Those with activity-limiting disabilities showed lower participation rates in aerobic physical activity and strength exercises when compared with elders without disabilities (see Table 2). The percentage (35.97%) of elders without disabilities who participated in aerobic physical activity of more than 150 min per week was about 1.5 times higher than that of elders with disabilities (24.09%). Additionally, in strength exercises per week, elders with disabilities showed lower participation (0.39 ± 0.24 days) than those without disabilities (0.69 ± 0.60 days). Participation in strength training was 10.23% for those with disabilities and 17.9% for those without. The findings reveal that both types of elders, with and without disabilities, participated in resistance exercises less than the minimum of two days per week recommended by the ACSM and the American Heart Association [17].

Elders experience depression and stress, which is expressed as worry, gloom, helplessness, and worthlessness. Some of the most frequent mental health problems experienced in old age occur due to physical changes, along with changes in social roles and support systems [30]. Among elders, depression and stress show an association with poorer physical health [31], and it has been reported that elders with disabilities have 2.68 times more depression than healthy elder individuals [32]. Regarding the mental health variable of activity-limiting disabilities, elders with disabilities were found to exhibit a higher risk of stress and depression compared to elders without disabilities. In the PHQ-9 test, those with disabilities showed a statistically higher depression-related index (6.12 ± 5.81) compared to elders without disabilities (2.36 ± 3.64), indicating that elders without disabilities were not as prone to depression as those with disabilities.

An active lifestyle is associated with health-related fitness and functionality of independence as well as the quality of life among individuals with disabilities [33]. However, according to the U.S. Department of Health and Human Services 2010 Report, 56% of people with disabilities are unable to participate in any leisure-time physical activity due to their activity limitations, and this has affected their quality of life [34]. In this study, elders with disabilities faced greater risk in self-managing their daily lives besides being more prone to anxiety/depression compared to elders without disabilities. Thus, removing the various barriers that possibly hinder the participation of elders with disabilities in physical activity programs should be considered to improve their quality of life [35].

This study used the KNHANES medical categorization of activity-limiting disabilities into 24 categories to investigate the association of a specific medical category with physical activity, mental health, and quality of life among elders with disabilities. Based on these categories, no significant differences were seen in the physical activity and mental health domains. However, there were significant differences in the quality-of-life domain of motor ability and pain/discomfort. This was consistent with a study on patients with chronic diseases, such as those with hypertension and arthritis, whose quality of life was poor [36]. While some differences were highlighted in terms of the impact of medical categories on quality of life, the post-hoc test revealed that they were not significant. This may be associated with the small sample sizes in each medical category. Thus, significant differences among the elderly, in terms of medical categories, were not found in this study.

This study has several limitations. First, physical activity was not measured objectively, but rather using a self-reported questionnaire. However, the questionnaire’s reliability and validity have been verified as it was used according to national standards [24]. Second, this study may not sufficiently reflect the characteristics of activity-limiting disabilities associated with different medical categories, as the sample sizes of some medical categories were limited, which also limited our statistical analyses. Third, the KNHANES has no information on the number of people with two, three, four, or more medical conditions. Fourth, the KNHANES provided “old age” as an option for one of the medical categories even though participants were under 80 years old. Hence, future studies should be conducted to address these various limitations.

## 5. Conclusions

Comparing the physical activity, mental health, and quality of life of 1504 elders with and without activity-limiting disabilities showed significant differences in (a) aerobic physical activity participation rates and number of days of doing strength exercises in the physical activity domains; (b) stress and depression in the mental health domains; and (c) all variables (motor ability, self-management, daily life, pain/discomfort, and anxiety/depression) in the quality-of-life domains between the elderly with and without disabilities. In terms of medical category, there were no differences in the physical activity and mental health domains, but there were differences in motor ability and pain/discomfort in the quality-of-life domain. From these results, it is possible to grasp the levels of physical activity, mental health, and quality-of-life status of the elderly with an activity-limiting disability status. This can serve as basic data for further studies on activity limitations among the elderly.

## Figures and Tables

**Table 1:** General participant characteristics (*n* = 1504).

Characteristics		Mean ± Standard Deviation or *n* (%)
Age (years)		72.79 ± 5.01
Gender	Male	663 (44.1%)
	Female	841 (55.9%)
Marital status	Married	1495 (99.4%)
	Single	9 (0.6%)
Marital condition	Residence with spouse	1002 (66.6%)
	Separation from spouse	8 (0.5%)
	Separation by death from spouse	428 (28.5%)
	Divorced from spouse	57 (3.8%)
	Single	9 (0.6%)
National basic livelihood recipient status	Receipt	165 (11.0%)
	No receipt	1338 (88.9%)
	Unknown	1 (0.1%)
Personal income	Upper 25%	373 (24.7%)
	Upper 25∼50%	371 (24.5%)
	Upper 50∼75%	374 (24.7%)
	Upper 75∼100%	386 (25.5%)

**Table 2:** Differences in physical activity, mental health, and quality of life in elders with and without disabilities (*n* = 1504).

Variables		Elders with Disability (*n* = 303)	Elders without Disability (*n* = 1201)	*p* Value
Physical activity	Participation in aerobic physical activity (%)	0.24 ± 0.43	0.36 ± 0.48	0.003 **
	Number of days for strength exercise (days/week)	0.39 ± 0.24	0.69 ± 0.60	<0.001 ***
	Number of days for walking each week (days/week)	2.66 ± 1.78	3.51 ± 1.82	<0.001 ***
Mental health	Stress (points)	2.79 ± 0.94	3.20 ± 0.86	<0.001 ***
	Depression (points)	6.12 ± 5.81	2.36 ± 3.64	<0.001 ***
Quality of life	Motor ability (points)	1.75 ± 0.52	1.33 ± 0.62	0.007 **
	Self-management (points)	1.29 ± 0.49	1.10 ± 0.56	0.013 *
	Daily life (points)	1.54 ± 0.55	1.20 ± 0.58	0.018 *
	Pain/discomfort (points)	1.85 ± 0.79	1.38 ± 0.70	<0.001 ***
	Anxiety/depression (points)	1.41 ± 0.72	1.16 ± 0.58	0.021 *

Data are presented as mean ± standard deviation; * *p* < 0.05, ** *p* < 0.01, *** *p* < 0.001; test by analysis of covariance, adjusted for age, sex, marital status, notional basic livelihood, recipient status, and personal income, was performed.

**Table 3:** Medical category frequencies among elders with disabilities.

Disability Type	*n* (%)	Disability Type	*n* (%)
Fracture/articulation injury	12 (2.5%)	Hearing problem	19 (3.9%)
Other injuries	13 (2.7%)	Dementia	5 (1.0%)
Arthritis/rheumatism	83 (17.1%)	Depression, anxiety, emotional problems	15 (3.1%)
Heart disease	10 (2.1%)	Mental retardation	1 (0.2%)
Breathing problems, lung disease, asthma	23 (4.8%)	Obesity	0 (0%)
Stroke	19 (3.9%)	Old age	26 (5.4%)
Diabetes	14 (2.9%)	Renal failure	2 (0.4%)
Hypertension	5 (1.0%)	Dizziness	17 (3.5%)
Back/neck problems	96 (19.8%)	Gastrointestinal disturbances	10 (2.1%)
Cancer	7 (1.4%)	Knee/leg pain (except for arthritis)	34 (7.0%)
Tooth and dental disease	6 (1.2%)	Headache	7 (1.4%)
Vision problems	18 (3.7%)	Other	42 (8.7%)

**Table 4:** Differences in physical activity, mental health, and quality of life among the elderly based on medical category.

Disability type	Physical Activity			Mental Health		Quality of Life				
	Aerobic Physical Activity Participation	Number of Days for Strength Exercise	Number of Days of Walking per Week	Stress	Depression	Motor Ability *	Self-Management	Daily Life	Pain/Discomfort *	Anxiety/Depression
Fracture/articulation injury	0.07 ± 0.03	1.18 ± 0.59	3.49 ± 2.82	2.78 ± 0.71	7.20 ± 7.48	1.90 ± 0.51	1.57 ± 0.51	1.91 ± 0.66	2.24 ± 0.74	1.49 ± 0.51
Other injuries	0.32 ± 0.49	1.38 ± 1.38	2.53 ± 2.51	3.24 ± 0.60	2.91 ± 4.44	1.76 ± 0.43	1.52 ± 0.65	1.39 ± 0.52	1.67 ± 0.47	1.23 ± 0.43
Arthritis/rheumatism	0.20 ± 0.42	1.32 ± 1.11	3.17 ± 2.68	2.70 ± 0.94	7.08 ± 5.75	1.91 ± 0.50	1.36 ± 0.52	1.64 ± 0.54	2.07 ± 0.67	1.46 ± 0.64
Heart disease	0.20 ± 0.45	0.81 ± 0.64	3.81 ± 2.79	2.72 ± 0.69	6.38 ± 4.28	1.49 ± 0.52	1.29 ± 0.47	1.69 ± 0.69	1.60 ± 0.69	1.39 ± 0.51
Breathing problems, lung disease, asthma	0.13 ± 0.35	0.91± 0.62	3.00 ± 2.08	2.72 ± 0.92	6.38 ± 4.75	1.51 ± 0.58	1.22 ± 0.40	1.51 ± 0.50	1.83 ± 0.70	1.50 ± 0.58
Stroke	0.26 ± 0.44	1.48 ± 1.41	2.94 ± 2.43	2.74 ± 1.04	5.26 ± 6.09	1.94 ± 0.61	1.54 ± 0.70	1.77 ± 0.65	1.84 ± 0.76	1.43 ± 0.62
Diabetes	0.15 ± 0.35	1.00 ± 0.00	2.39 ± 2.46	2.55 ± 1.10	8.68 ± 6.59	1.82 ± 0.35	1.27 ± 0.60	1.79 ± 0.43	2.13 ± 0.63	1.49 ± 0.53
Hypertension	0.79 ± 0.46	1.81 ± 1.29	5.00 ± 1.59	2.43 ± 0.90	8.38 ± 7.20	1.59 ± 0.54	1.18 ± 0.44	1.78 ± 0.45	2.00 ± 0.70	1.20 ± 0.46
Back/neck problems	0.24 ± 0.44	1.36 ± 1.16	3.39 ± 2.80	2.72 ± 0.93	6.88 ± 6.12	1.82 ± 0.43	1.33 ± 0.51	1.60 ± 0.56	2.04 ± 0.61	1.43 ± 0.58
Cancer	0.42 ± 0.56	2.43 ± 2.45	5.42 ± 3.20	2.85 ± 1.05	7.41 ± 6.45	1.56 ± 0.53	1.28 ± 0.50	1.00 ± 0.00	1.28 ± 0.50	1.43 ± 0.55
Tooth and dental disease	0.18 ± 0.43	1.00 ± 0.88	2.19 ± 1.83	2.66 ± 1.07	1.23 ± 0.49	2.00 ± 0.62	1.31 ± 0.81	1.65 ± 0.81	2.15 ± 0.99	1.66 ± 0.51
Vision problems	0.21 ± 0.32	1.67 ± 1.64	3.77 ± 2.75	2.86 ± 1.01	6.48 ± 5.83	1.60 ± 0.49	1.15 ± 0.37	1.55 ± 0.50	1.88 ± 0.75	1.25 ± 0.44
Hearing problems	0.38 ± 0.48	1.15 ± 0.68	4.96 ± 2.98	2.93 ± 0.98	4.76 ± 4.75	1.42 ± 0.56	1.10 ± 0.31	1.25 ± 0.55	1.57 ± 0.68	1.35 ± 0.59
Dementia	0.00 ± 0.00	0.78 ± 0.83	3.20 ± 2.51	2.42 ± 1.33	4.43 ± 3.27	1.79 ± 0.82	1.58 ± 0.90	1.81 ± 0.44	1.79 ± 0.44	1.59 ± 0.88
Depression, anxiety, emotional problems	0.29 ± 0.48	0.92 ± 0.45	3.48 ± 2.99	2.22 ± 0.93	10.51 ± 0.21	1.59 ± 0.50	1.25 ± 0.44	1.41 ± 0.52	1.66 ± 0.89	1.91 ± 0.69
Mental retardation	0.00 ± 0.00	1.00 ± 0.00	5.00 ± 0.00	2.00 ± 0.00	3.00 ± 0.00	2.00 ± 0.00	2.00 ± 0.00	2.00 ± 0.00	2.00 ± 0.00	1.00 ± 0.00
Obesity	-	-	-	-	-	-	-	-	-	-
Old age	0.17 ± 0.38	1.00 ± 0.68	2.78 ± 2.67	2.96 ± 1.00	6.36 ± 5.08	1.91 ± 0.41	1.34 ± 0.54	1.84 ± 0.53	2.26 ± 1.48	1.87 ± 1.57
Renal failure	0.00 ± 0.00	0.51 ± 0.72	2.00 ± 1.42	2.00 ± 1.40	0.86 ± 1.05	1.49 ± 0.68	1.00 ± 0.00	1.50 ± 0.72	1.49 ± 0.72	1.50 ± 0.70
Dizziness	0.34 ± 0.50	1.07 ± 0.74	4.23 ± 2.67	2.71 ± 0.86	7.87 ± 5.98	1.93 ± 0.19	1.45 ± 0.50	1.64 ± 0.50	1.93 ± 0.55	1.46 ± 0.61
Gastrointestinal disturbances	0.32 ± 0.50	2.11 ± 2.09	4.00 ± 3.16	3.13 ± 2.15	7.22 ± 5.23	1.91 ± 0.32	1.19 ± 0.42	1.59 ± 0.51	2.00 ± 0.00	1.39 ± 0.51
Knee/leg pain (except for arthritis)	0.19 ± 0.41	1.45 ± 1.32	3.45 ± 2.78	2.95 ± 1.41	8.16 ± 5.96	1.80 ± 0.62	1.27 ± 0.51	1.69 ± 0.58	2.17 ± 0.57	1.40 ± 0.55
Headache	0.15 ± 0.39	2.00 ± 1.93	4.00 ± 3.05	2.86 ± 0.92	6.18 ± 5.03	1.41 ± 0.51	1.13 ± 0.37	1.30 ± 0.50	2.00 ± 0.57	1.30 ± 0.50
Other	0.28 ± 0.48	1.32 ± 1.01	4.13 ± 2.71	2.77 ± 0.80	7.20± 5.91	1.58 ± 0.51	1.30 ± 0.51	1.54 ± 0.49	1.71 ± 0.64	1.36 ± 0.49

Data are presented as mean ± standard deviation; * *p* < 0.05; test by analysis of covariance, adjusted for age, sex, marital status, notional basic livelihood, recipient status, and personal income, was performed.
